# The Association between Working Hours with Vigilance and Executive Function of Intensive Care Unit Nurses

**DOI:** 10.1155/2023/3770404

**Published:** 2023-12-09

**Authors:** Meng-Juan Jing, Hao Li, Chun-Peng Li, Xiao-Jing Wei, Wei-Quan Lin, Shi-Chao Zhu, Yu-Lin Xu, Li-Ming Li

**Affiliations:** ^1^Department of Critical Care Medicine, Henan Provincial People's Hospital, Zhengzhou University People's Hospital, Henan Key Laboratory for Nursing, Zhengzhou Key Laboratory of Critical Care Nursing, Zhengzhou, Henan, China; ^2^Department of Basic Public Health, Center for Disease Control and Prevention of Guangzhou, Guangzhou, China; ^3^Department of Cardiovascular Medicine, The First Affiliated Hospital of Zhengzhou University, Zhengzhou, Henan, China

## Abstract

**Background:**

Vigilance and executive functions are integral to nursing practice. Prolonged working hours are associated with heightened fatigue and increased nursing errors. However, the impact of work duration on the vigilance and executive function of ICU nurses remains unexplored.

**Objective:**

This study aims to elucidate the association between ICU nurses' working hours, vigilance, and executive function.

**Design:**

A cross-sectional study. *Setting*. Intensive care medicine department of a tertiary hospital in Zhengzhou, China. *Participants*. A total of 51 registered nurses who participated in 12 h shifts in the ICU completed the survey.

**Methods:**

E-prime software was employed to develop four test tasks to measure the vigilance and executive functioning of ICU nurses. The test was performed before the start of the shift and after 4, 8, and 12 h.

**Results:**

The analysis revealed no statistically significant differences in the response time of vigilance for ICU nurses across shifts (*p*=0.503) or working hours (*p*=0.078). However, a significant difference existed in lapses across working hours (*p*=0.005), significantly increasing after 8 and 12 h. The analysis indicated a significant difference in Flanker effect size across different working hours (*p*=0.035). The analysis revealed no significant differences in the switch cost (*p*=0.200) or response accuracy (*p*=0.479) of the task switching across working hours. The response accuracy for the 2-back task differed significantly (*p*=0.003) across working hours.

**Conclusion:**

Limited evidence demonstrated that vigilance and specific aspects of executive functioning (inhibitory control and working memory) of ICU nurses were negatively correlated with the duration of their work in a real clinical setting. Furthermore, no vigilance and executive function differences were identified between day and night shifts. *Implications for Nursing Management*. Nursing administrators should reconsider scheduling 12 h shifts, shortening shifts, or implementing short rest periods to reduce fatigue and cognitive load. In addition, flexible scheduling and rationalizing the order of work may help reduce the possible risks associated with prolonged work.

## 1. Background

It is well known that ICU patients have complex conditions, imparting a high workload on ICU nurses as they are responsible for observing patient condition changes and completing various nursing practices and records. Significant physical and mental demands pose significant challenges for ICU nurses [1]. ICU nurses must maintain high vigilance and executive function (EF) to ensure patient safety. However, research in psychology and neuroscience suggests that the human brain has limited cognitive resources, such as the attention span and working memory capacity. Multitasking and attentional interference can slow response times and increase the likelihood of errors [2, 3]. When ICU nurses lose vigilance in clinical settings, they may fail to detect patient condition changes, resulting in treatment delays. Furthermore, as EF declines, nurses may become more susceptible to interruptions, potentially leading to nursing errors. Therefore, vigilance and EF are crucial for patient safety, and further research is being conducted in these areas.

Vigilance is the ability of an individual to maintain attention to tasks over time. Initially, vigilance research focused primarily on high-concentration occupations such as pilots and drivers [4]. Poor driver vigilance results in reduced sustained attention and increased risk of human error, which can have serious consequences. There is growing recognition of the importance of vigilance for the safety of both caregivers and patients. Monitoring and responding immediately to patient condition changes are fundamental nursing responsibilities, making vigilance at the core of nursing practice [5]. Subsequent nursing actions cannot be performed without constant patient observation.

Executive function is the ability of an individual to control and regulate complex cognitive tasks, which is a higher-order cognitive function [6]. Zelazo and Müller classified EFs into two types based on brain imaging: hot executive function and cold executive function [7]. Hot executive function involves emotional engagement and flexible evaluation of rewarding and stimulating tasks, whereas cold executive function lacks emotional involvement and is often characterized by logical and abstract thinking [7]. Numerous studies have been conducted on cold executive function, and the widely used model divides it into three aspects: inhibitory control ability, shifting, and working memory, each closely related to nursing performance [8]. Compared to other professions, nurses require multitasking skills and the ability to quickly shift attention and maintain memory between patients and different events. In nursing practice, nurses must carefully plan their workflow to avoid omissions or errors, a process requiring high executive capacity.

Vigilance and EF of nurses are not solely affected by disruption of the day-night sleep schedule but also the length of working hours, which should not be overlooked. In clinical practice, nurses routinely work 8 or 12 h shifts. Although a 12 h shift may reduce the number of shifts and commuting time while also allowing for more continuous patient care, it is believed that prolonged periods of uninterrupted work are associated with increased fatigue and poor performance [9]. A study demonstrated that nurses working 12.5 h or more had more than three times the risk of errors than those who worked less than 8 h [10]. Furthermore, a systematic review reported that nurses working 12 h shifts had a higher incidence of self-reported errors than those working 8 h shifts, posing a potential threat to patient safety [11].

Based on a comprehensive review of the existing literature, we found that vigilance and EF are impaired among day-night shift-working nurses. However, most studies attribute decreased vigilance and EF to shift nurses' circadian disruptions [12, 13]. The relationship between working hours, nurses' vigilance and EF has been widely underestimated. In addition, studies on the ICU nurse population are still lacking, and limited data on vigilance and EF of Chinese nurses are scarce. Furthermore, our study may be the first to assess EF through three computerized tasks based on a theoretical model, which can contribute to further understanding of impaired EF in nurses [8]. Given these gaps in the literature, the present study used a computerized task to investigate the impact of working hours on the vigilance and executive function of ICU nurses. This study aimed to collect empirical evidence to help managers make informed decisions regarding scheduling practices.

The study is designed based on two hypotheses:

  Hypothesis 1. ICU nurses' vigilance and EF were negatively correlated with work hours.
  Hypothesis 2. Nurses exhibit lower levels of vigilance and EF during night shifts than day shifts.

## 2. Methods

### 2.1. Design

This study used a cross-sectional design with E-prime software for task programming and data collection. The practice session occurred from February 13 to February 26, 2023, and data collection was performed from March 6 to March 26, 2023. To ensure a thorough understanding of the task rules, all participants were required to practice for a minimum of 20 min with at least 80% accuracy during the practice session.

### 2.2. Participants

This study was conducted in the Department of Critical Care Medicine at Henan Provincial People's Hospital, Zhengzhou, China. The work schedule (day-night-off night and rest) aligns with hospital regulations and rules, ensuring continuous 24 h service for patient care, as is typical in ICUs in Chinese hospitals. Day-shift nurses worked from 8 a.m. to 8 p.m., and night-shift nurses worked from 8 p.m. to 8 a.m. Registered nurses working 12 h shifts were eligible for inclusion in the study. Individuals who had taken psychotropic drugs in the last three months or were pregnant were excluded. Considering that there were no relevant data in China before, the sample size was selected based on similar studies [14, 15]. A total of 52 nurses participated in the practice sessions. However, one nurse dropped out due to other training, resulting in a final dataset of 51 nurses.

### 2.3. Measures

**Figure 1:**
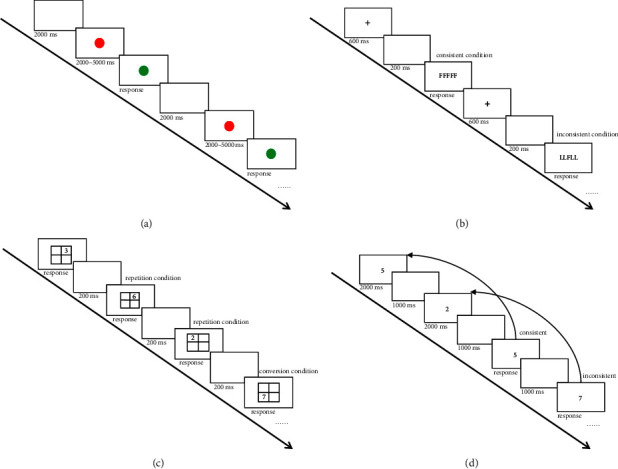
Experimental paradigm: (a) psychomotor vigilance task, (b) Flanker task, (c) task switching, and (d) 2-back task.

The test task for the present study was developed using E-Prime 2.0, and the experimental paradigm is illustrated in Figure 1. The task was performed on a 14-inch laptop connected to an external 21.5-inch monitor with a 1920 × 1080 pixel screen resolution and a refresh rate of 75 Hz. The test was conducted in a quiet room approximately 50 m from the ward, with participants seated approximately 60 cm in front of a computer screen. Participants completed eight tests: before (day and night) shifts (T0), after 4 h (T1), after 8 h (T2), and after 12 h (T3) of work. Because clinical practice is unpredictable, data collection was permitted to be completed within 30 min before and after the scheduled time.

#### 2.3.1. Vigilance

The present study used a 3 min version of the psychomotor vigilance task (PVT) to evaluate nurses' vigilance. PVT is widely recognized as the gold standard for assessing behavioral vigilance and attention due to its high sensitivity to sleep deprivation [16]. Results of extensive experiments on PVT performance have demonstrated that the task is capable of capturing the effects of sleep loss on the stability of sustained attention and that it can reliably reveal the accumulation of cumulative state instability in chronic sleep loss. The reliability of the test has been reported to be 0.888, falling into the standardized “almost perfect” range for a measurement assay [17]. The test-retest reliability in our study was between 0.767 and 0.879. A red circle is displayed in the center of the screen in the PVT. When the color changes from red to green (randomly occurring between 2000 and 5000 ms) participants must press the “L” key as quickly as possible. The red circle reappears two seconds after the response, repeating the process. If no response was made within five seconds, the stimulus ended, and the timer for the subsequent stimulus was reset. A response time (RT) of less than 100 ms was excluded from the analysis, while more than 500 ms were recorded as lapses [16]. Longer mean reaction times or greater lapses indicated a lack of nurse vigilance.

#### 2.3.2. Executive Function

The Flanker, Stroop, and Simon tasks are commonly used to assess inhibitory control ability [18]. The letter version of the Flanker task was used in the present study to measure the inhibitory control ability of nurses. The validity of the task has been reported to be ≥0.73. The reliability of the task has been reported to be between 0.86 and 0.92 [19]. The test-retest reliability in our study was between 0.773 and 0.866. Four letter strings (“FFFFF,” “LLLLL,” “LLFLL,” and “FFLFF”) were randomly presented in the center of the screen. Participants were instructed to respond to the middle letter (e.g., pressing the “F” key if the middle letter was an “F”) as quickly as possible. Both consistent and inconsistent conditions were randomly and equally presented. The Flanker effect size (FES) and incorrect responses (IR) for each subject were recorded during the task. The FES represents the difference in RT between consistent and inconsistent conditions. A smaller effect size indicates better inhibitory control ability.

Task switching was used to measure shifting [20, 23]. The high validity of the task has been demonstrated in extensive research. The reliability of the task has been reported to be between 0.859 and 0.910 [9, 22]. The test-retest reliability in our study was between 0.740 and 0.862. In this task, a 2 × 2 grid was displayed in the middle of the screen, and a number (randomly ranging from 1 to 9, excluding 5) was presented within the grid. The participants were asked to judge the size or parity of the number based on their location within the grid. They had to determine whether the number was greater or less than five if it appeared in the upper grid and its parity if it appeared in the lower grid. They were instructed to press the “F” key if the result was less than five or odd and the “L” key otherwise. The switch cost (SC), which is the difference in reaction time between the conversion and repetition conditions, and IR were recorded during the task. Lower SCs or higher accuracy suggest better cognitive flexibility.

A digital 2-back task was used to assess the working memory. The N-back task is a classical working memory paradigm frequently used in working memory-related studies [23, 24]. The validity of the test to measure working memory has been reported in several studies [25–28]. The split-half reliabilities of the test have been reported to be between 0.802 and 0.824 [29]. The test-retest reliability in our study was between 0.670 and 0.792. A series of stimuli were presented sequentially in the 2-back task, and participants had to judge whether the current number matched the number presented two stimuli earlier. They used the “F” key for a consistent match and the “L” key for inconsistency. The task recorded RT and IR. A shorter reaction time or higher accuracy indicates working memory.

### 2.4. Data Analysis

IBM SPSS version 26.0 was used to analyze the data. The demographic characteristics of ICU nurses were described using frequencies, percentages, mean/medians, standard deviation (SD), and range. MANOVA was used for the statistical inference of normally distributed data. Non-normally distributed data are presented as median (interquartile range), and nonparametric tests and generalized estimating equations (GEEs) were used for statistical inference. The statistical significance level was set at *p* < 0.05.

### 2.5. Ethical Approval

This study adhered to the 2000 revision of the Helsinki Declaration and followed the ethical standards outlined by the responsible committee on human experimentation. The study protocol was approved by the Institutional Review Board of Henan Provincial People's Hospital (approval date: 20220620). All participants provided written informed consent before they participated in the study.

## 3. Results

### 3.1. Demographic Characteristics of the Sample

Table 1 summarizes the demographic characteristics of ICU nurses. Most participants (70.6%) were females, and their mean age was 29.10 years, SD = 4.15. Over half of the participants (66.7%) had worked in the ICU for more than five years. All participants reported no symptoms of discomfort and were not colorblind. In addition, they reported no preference for coffee or tea at work.

### 3.2. Vigilance

**Figure 2:**
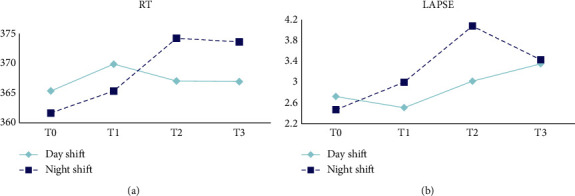
Changes of RT and lapse of PVT.

Table 2 and Figure 2 reveal the changes in vigilance, RT, and lapse among ICU nurses. MANOVA revealed no statistically significant difference in RT for ICU nurses across working hours (*P*_sphericity_ = 0.686, *F* = 2.320, *p*=0.078, *η*^2^ = 0.044) and shifts (*F* = 0.456, *p*=0.503*η*^2^ = 0.009). However, lapse occurrence varied significantly across working hours (Wald*χ*^2^ = 12.844, *p*=0.005), with a significant increase observed after 8 and 12 h.

### 3.3. Executive Function

#### 3.3.1. Inhibitory Control Ability

**Figure 3:**
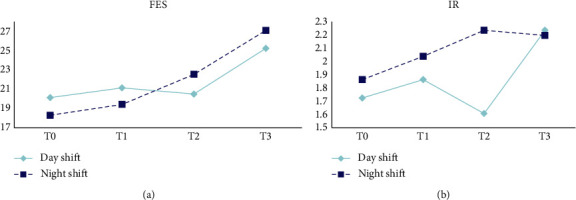
Changes of FES and IR of Flanker test.

The changes in FES and IR among ICU nurses are presented in Table 3 and Figure 3. MANOVA indicated a significant difference in the FES across different working hours (*P*_sphericity_ = 0.359, *F* = 2.943, *p*=0.035, *η*^2^ = 0.056). However, the IR of the Flanker task did not differ significantly among different working hours (Wald*χ*^2^ = 2.747, *p*=0.432) and shifts (Wald*χ*^2^ = 1.477, *p*=0.224).

#### 3.3.2. Shifting

**Figure 4:**
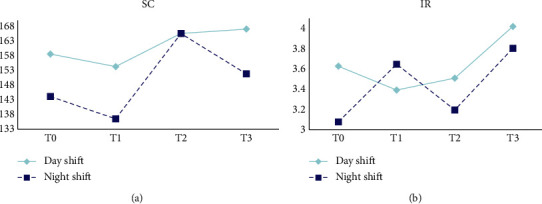
Changes of SC and IR of task switching.

Table 4 and Figure 4 represent the changes in SC and IR among the ICU nurses. MANOVA revealed no significant differences in the SC of the task switching among different working hours (*P*_sphericity_ = 0.093, *F* = 1.568, *p*=0.200, *η*^2^ = 0.030) and shifts (*F* = 2.768, *p*=0.102, *η*^2^ = 0.052). In addition, the generalized estimating equation indicated no significant differences in IR among different working hours (Wald*χ*^2^ = 2.481, *p*=0.479) and shifts (Wald*χ*^2^ = 0.588, *p*=0.443).

#### 3.3.3. Working Memory

**Figure 5:**
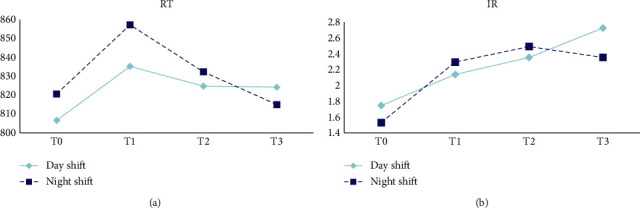
Changes of RT and IR of 2-back.

The changes in RT and IR to the 2-back task among the ICU nurses are presented in Table 5 and Figure 5. Repeated MANOVA revealed no significant difference in RT for the 2-back task among different working hours (*P*_sphericity_ = 0.486, *F* = 1.483, *p* = 0.221, *η*^2^ = 0.029) and shifts (*F* = 0.309, *p* = 0.581, *η*^2^ = 0.006). However, there was a significant difference in IR between working hours (Wald*χ*^2^ = 13.916, *p* = 0.003) for the 2-back task.

## 4. Discussion

Although previous studies have reported the impact of shifts and 12 h work on nurses' fatigue and vigilance, their findings remain contentious. Notably, the present study is possibly the first to investigate the vigilance and EF of Chinese ICU nurses in a real clinical setting. Furthermore, it may be the first to comprehensively assess EF using three computerized tasks based on a theoretical model. The present study contributes to our understanding of how consecutive 12 h rotating shifts affect the vigilance and EF of ICU nurses while providing research data from a different population to support the ongoing debate. There is limited evidence from our study that there is a negative correlation between vigilance and certain aspects of EF in ICU nurses and their working hours. Furthermore, our study revealed no significant differences in vigilance and EF between night and day shifts among nurses.

The findings revealed that the vigilance reaction time of ICU nurses did not differ significantly across different working hours (*p*=0.078), consistent with previous findings [14]. Although this result did not demonstrate statistical significance, we cannot conclude that the vigilance of ICU nurses is unaffected by working hours. Several factors may have contributed to these findings. First, given the busyness of clinical practice, we used a 3 min version of the PVT task rather than 5 or 10 min. Only 30 stimuli were presented in our task, which may have limited our ability to capture attention and effectively detect fatigue. Second, we set the lapse threshold to 500 ms. However, this threshold may have been relatively low for our PVT task. This is because, unlike some PVT tasks, our task did not present the RT after each stimulus presentation. This could have resulted in less positive feedback from our participants, resulting in longer RTs. Responses exceeding 500 ms were considered lapses and excluded from the average RTs analysis, which may have influenced our findings. Furthermore, the small sample size introduced sampling errors, which should not be overlooked as a contributing factor.

Another important observation indicator in the PVT task is the number of lapses, which reflects an individual's ability to maintain attention. Our study revealed that regardless of the day or night shift, the number of lapses among ICU nurses increased as the duration of work increased. This suggests a negative relationship between nurses' attention and work duration, implying that their vigilance decreases as nurses work longer shifts. This is unsurprising, given that nurses work 12 h shifts and accumulate significant fatigue and sleep debt. Nurses experience increased drowsiness and a gradual decline in vigilance as their shift lengthens [30].

Furthermore, pairwise comparison results indicate that the number of lapses among nurses at T2 and T3 was significantly higher than at T0 and T1. This suggests that ICU nurses consistently have low vigilance after 8 h. Therefore, we advise managers to reconsider the potential risks to patient safety associated with the current 12 h shift schedule in the ICU.

Most studies have indicated that night-shift nurses have lower and more unstable vigilance than day-shift nurses [31–33]. Although there is no interaction effect between working hours and shift for nurses' RT (*p*=0.069) and the number of lapses (*p*=0.216) and no within-group differences in terms of shift, the trend in changes in nurses' RT and the number of lapses (Figure 2) seems to suggest a similar pattern of instability in vigilance during night shifts. This is most likely due to circadian rhythm adaptation, as even experienced ICU nurses exhibit higher drowsiness during night shifts than during day shifts.

In summary, we conclude that the vigilance of ICU nurses decreases with increasing work duration, with more pronounced vigilance impairment after 8 h of work. Furthermore, the vigilance of ICU nurses during night shifts appeared to be more susceptible to work duration.

Another focus of our study was on the EF of nurses. Impaired EF among nurses in clinical settings can lead to serious medical errors. Inhibitory control is an important component of EF [34]. Individuals with poor inhibitory control may be easily distracted, have difficulty maintaining attention, and have difficulty suppressing impulsive behaviors. Nurses frequently encounter various distractions and stimuli during clinical work, such as noise from equipment and colleagues' interactions. Nurses with stronger inhibitory control can approach their work goals more rationally and efficiently. The findings of the present study reveal a significant difference in the FES of ICU nurses at different work durations (*p*=0.035), implying that the inhibitory control of nurses decreases as work duration increases. This is mainly due to fatigue and stress caused by continuous work, which affect the activity of the prefrontal cortex, which is involved in higher-order cognitive processing. Therefore, the inhibitory control of nurses is compromised.

Shifting refers to the ability of an individual to switch behaviors flexibly in different contexts and is equally important for nurses. ICU nurses face other patients and situations at work, requiring them to adjust their care plans and behaviors based on the needs and conditions of their patients. The present study revealed no differences in cognitive flexibility among nurses with different work durations. This could be due to two factors. First, the participants included in our study are relatively young (29.10 ± 4.08), and younger individuals often have an advantage in maintaining cognitive flexibility. Second, research suggests that learning and training can improve an individual's cognitive flexibility [35, 36]. Over half of the participants in the present study had more than five years of ICU work experience, which may have cultivated their flexibility and better adaptation to various cognitive tasks.

Working memory capacity is an important factor for nurses. In clinical practice, nurses must constantly grasp and remember patients' conditions, treatment progress, nursing measures, job tasks, and operational steps. These pieces of information must be acquired, processed, promptly, and accurately recalled. Consistent with a previous study [37], we identified statistically significant differences in the error rates of the 2-back task among participants with varying work durations (*p*=0.003). This finding indicates a negative correlation between ICU nurses' working memory and work duration. Long periods of continuous work may deplete nurses' cognitive resources. As work duration increases, nurses must handle more tasks and information, which may exceed their working memory capacity, resulting in information loss or memory forgetting. Although there were no differences in working memory reaction times among nurses with different work durations in this study, we observed a slight increase in working memory reaction times at T1. We infer that this may be associated with the workload. Generally, T1 (12:00 p.m. or 12:00 a.m.) is one of the busiest times in clinical work, and nurses face a higher memory load when multitasking and memorizing multiple pieces of information simultaneously. This may affect the speed of memory retrieval during testing.

In summary, we believe that certain aspects of EF (inhibition control, working memory) of ICU nurses decline as work duration increases. Furthermore, ICU nurses have similar EF levels during night shifts compared to day shifts.

## 5. Limitations

The present study has several limitations. First, smoking and coffee consumption may have influenced the test results. However, only two participants in our study smoked, and none drank coffee at work. Therefore, we did not include these factors in our statistical analysis, which may have limited the extrapolation of our study. Second, because we only tested nurses from one ICU, our sample size was likely to be small, which could have resulted in sampling errors. Moreover, due to the small sample size, we did not conduct subgroup analyses based on gender or the menstrual cycle. Finally, despite including a practice phase to minimize practice effects, they may still exist to some extent.

## 6. Conclusions

Nurses serve as the “eyes and hands” of the ICU, monitoring and intervening in patients' health. The vigilance and EF of nurses are crucial for patient safety. Although longer shifts have been praised for increasing productivity and lowering staffing costs, our study examined ICU nurses working 12 h shifts using objective computer tasks. There is limited evidence showing that vigilance and some aspects of EF in ICU nurses are negatively correlated with the duration of their shifts in a real clinical setting. Furthermore, no vigilance or EF differences were identified between day and night shifts.

## 7. Implications for Nursing Management

To reduce fatigue and cognitive load, nursing administrators should reconsider scheduling 12 h shifts, shortening shifts, or implementing short rest periods. Such adjustments are beneficial and necessary for nurses' well-being and patient safety. Flexible work may be another approach to address this problem. Allowing nurses to adjust their working hours according to their habits and status may help staff perform at their best and improve the quality and efficiency of their work. In addition, managers should pay attention to staff fatigue, especially after working for more than 8 h. Managers can also adjust the order of work, such as scheduling complex tasks at the beginning of the work as much as possible, while simple executive tasks are scheduled later, which may reduce the possible risks associated with prolonged work.

## Figures and Tables

**Table 1:** Demographic characteristics (*N* = 51).

Characteristics	*N*	%
Age		
<30	24	47.1
≥30	27	52.9
Gender		
Male	15	29.4
Female	36	70.6
Marital status		
Unmarried	24	47.1
Married	27	52.9
Working experience in ICU (years)		
<5	17	33.3
5–10	23	45.1
>10	11	21.6
Professional title		
Primary titles	28	54.9
Secondary titles and above	23	45.1

**Table 2:** Comparison of vigilance at different working hours (*N* = 51).

Variables	*T*0		*T*1		*T*2		*T*3		Statistical value (*p*)		
	Day	Night	Day	Night	Day	Night	Day	Night	Time	Shift	Interaction effect
RT	365.38 ± 33.12	361.68 ± 39.73	369.87 ± 39.09	365.36 ± 41.85	367.05 ± 37.88	374.21 ± 46.33	366.94 ± 37.80	373.60 ± 39.68	2.320^a^ (0.078)	0.456^a^ (0.503)	2.516^a^ (0.069)
Lapse	2 (1, 4)	1 (0, 4)	2 (1, 4)	1 (0, 4)	2 (1, 5)	3 (1, 7)	2 (1, 5)	3 (1, 5)	12.844^b^ (0.005)	1.699^b^ (0.203)	4.460^b^ (0.216)

*Note*. ^a^*F*, ^b^Wald*χ*^2^.

**Table 3:** Comparison of inhibitory control ability at different working hours (*N* = 51).

Variables	*T*0		*T*1		*T*2		*T*3		Statistical value (*p*)		
	Day	Night	Day	Night	Day	Night	Day	Night	Time	Shift	Interaction effect
FES	20.14 ± 18.96	18.30 ± 18.64	21.15 ± 18.37	19.43 ± 20.47	20.50 ± 16.24	22.54 ± 25.47	25.23 ± 22.05	27.08 ± 19.99	2.943^a^ (0.035)	0.002^a^ (0.963)	0.270^a^ (0.847)
IR	1 (1, 2)	2 (1, 3)	2 (1, 3)	2 (1, 3)	1 (0, 2)	2 (0, 3)	2 (0, 3)	2 (1, 3)	2.747^b^ (0.432)	1.477^b^ (0.224)	1.856^b^ (0.603)

*Note*. ^a^*F*, ^b^Wald*χ*^2^.

**Table 4:** Comparison of shifting at different working hours (*N* = 51).

Variables	*T*0		*T*1		*T*2		*T*3		Statistical value (*p*)		
	Day	Night	Day	Night	Day	Night	Day	Night	Time	Shift	Interaction effect
SC	158.59 ± 103.22	144.15 ± 100.84	154.30 ± 118.83	136.54 ± 93.97	165.58 ± 109.07	165.56 ± 109.55	167.06 ± 114.10	151.85 ± 102.22	1.568^a^ (0.200)	2.768^a^ (0.102)	0.307^a^ (0.820)
IR	3 (1, 5)	3 (2, 4)	2 (2, 4)	3 (1, 5)	3 (2, 5)	3 (1, 4)	3 (2, 5)	3 (2, 4)	2.481^b^ (0.479)	0.588^b^ (0.443)	1.140^b^ (0.767)

*Note*. ^a^*F*, ^b^Wald*χ*^2^.

**Table 5:** Comparison of working memory at different working hours (*N* = 51).

Variables	*T*0		*T*1		*T*2		*T*3		Statistical value (*p*)		
	Day	Night	Day	Night	Day	Night	Day	Night	Time	Shift	Interaction effect
RT	806.54 ± 152.66	820.63 ± 185.72	835.22 ± 172.16	857.23 ± 221.35	824.73 ± 187.91	832.30 ± 180.74	824.21 ± 190.78	814.95 ± 199.31	1.483^a^ (0.221)	0.309^a^ (0.581)	0.259^a^ (0.855)
IR	1 (0, 2)	1 (0, 2)	2 (0, 3)	1 (0, 3)	2 (1, 3)	2 (1, 3)	2 (1, 4)	1 (0, 3)	13.916^b^ (0.003)	0.091^b^ (0.763)	1.944^b^ (0.584)

*Note*. ^a^*F*, ^b^Wald*χ*^2^.

## Data Availability

The data that support the findings of this study are available from the corresponding author upon reasonable request.

## Additional Points

*Tweetable Abstract*. Limited evidence has shown that vigilance and executive function of ICU nurses are negatively correlated with work duration.
